# Supplementary material for: Safety and Efficacy of Medical Cannabis in Fibromyalgia
Source: J Clin Med. 2019 Jun 5;8(6):807. doi: 10.3390/jcm8060807 (PMC6616435; doi:10.3390/jcm8060807)
Supplement: Supplementary file 1 [file jcm-08-00807-s001.pdf]

Supplementary table 1. Baseline characteristics of the patients stratified by response at six months follow-up.

**Variable**	**Non responders (n=156)**	**Responders (n=211)**	**P value**
Age (years), mean ± SD	51.9 (15.2)	53.7 (15.1)	0.252
Age groups, n (%)			
40>	35 (23.5)	40 (18.3)	0.195
40-60	73 (49.0)	101 (49.5)	
60<	41 (27.5)	70 (32.1)	
Females, n (%)	124 (83.2)	177 (81.2)	0.679
BMI, mean ± SD	27.0 (6.9)	26.5 (5.2)	0.389
Work status:			
Works regularly	24 (16.2)	35 (16.3)	0.807
Part time work	21 (14.2)	32 (14.9)	
Unemployed/retired	97 (65.5)	142 (66.0)	
Other	6 (4.1)	6 (2.8)	
Driving a car, n (%)	92 (61.7)	139 (63.8)	0.309
Approved monthly dosage of cannabis ≤ 20 grams, n (%)	127 (85.2)	201 (92.2)	0.039
Previous experience with cannabis, n (%)	70 (41.0)	96 (44.0)	0.798
Cigarettes smokers, n (%)	58 (38.9)	79 (36.2)	0.527
Number of regularly used medications, median (i.q range)	5.0 (3.0-7.0)	5.0 (3.0-8.0)	0.747
Number of regularly used medications for fibromyalgia, median (i.q range)	1.0 (0.0-2.0)	1.0 (1.0-3.0)	0.507
Treatment indication: Primary fibromyalgia, n (%)	116 (77.9)	167 (76.6)	0.558
Cancer, n (%)	15 (10.1)	20 (9.2)	
PTSD, n (%)	9 (6.0)	13 (6.0)	
Other, n (%)	9 (6.0)	18 (8.3)	

Years of chronic pain, median (i.q)	8.0 (3.0-12.0)	7.0 (3.2-14.0)	0.889
Type of pain: Daily, n (%)<br>Episodic , n(%)	128 (85.9)<br>21 (14.1)	192 (88.1)<br>26 (11.9)	0.634

BMI – body mass index, I.Q range – interquartile range, PTSD – post traumatic stress disorder

Supplementary table 2. Cannabis used by the patients.

Cannabis dominant species and classification	Strain name and Cannabinoids content (all contain CBN 0.9%)	Patients (%)	Utilization methods		
			Vaporizing or smoking	Oil	Solids
THC rich –Indica	Erez (THC 20%, CBD 1%)	173 (47.1)	73 (19.8)	39 (10.6)	6 (1.6)
	Dorit (THC 20%, CBD 1%)	62 (16.9)	25 (6.8)	0 (0.0)	1 (0.2)
	Or (THC 20%, CBD 1%)	16 (4.4)	16 (4.4)	0 (0.0)	0 (0.0)
	Eran Almog (THC 20%, CBD 1%)	9 (2.5)	9 (2.5)	0 (0.0)	0 (0.0)
	Barak (THC 20%, CBD 1%)	6 (1.6)	6 (1.6)	0 (0.0)	0 (0.0)
	Omer (THC 20%, CBD 1%)	5 (1.4)	5 (1.4)	0 (0.0)	0 (0.0)
	Tal (THC 20%, CBD 1%)	7 (1.9)	3 (0.8)	0 (0.0)	0 (0.0)
	Yasmin (THC 15%, CBD 0%)	8 (2.2)	3 (0.8)	0 (0.0)	1 (0.2)
THC rich – Sativa	Alaska (THC 20%, CBD 1%)	41 (11.2)	40 (11.0)	1 (0.2)	0 (0.0)
	Shira (THC 20%, CBD 1%)	7 (1.9)	7 (1.9)	0 (0.0)	0 (0.0)

**CBD rich – Indica**	Avidekel (THC 1%, CBD 18%)	110 (30.0)	20 (5.4)	31 (8.4)	4 (1.0)
**CBD rich – Sativa**	Raphael (THC 1%, CBD 20%)	19 (5.2)	15 (4.0)	0 (0.0)	2 (0.5)
**Equal THC & CBD – Indica**	Midnight (THC 10%, CBD 12%)	123 (33.5)	51 (13.9)	3 (0.8)	4 (1.0)
	Al-Na (THC 10%, CBD 5%)	6 (1.6)	0 (0.0)	0 (0.0)	0 (0.0)

Patient can use more than one strain.

THC – tetrahydrocannabinol, CBD – cannabidiol, CBN – cannabinol.

<u>Supplementary table 3</u>. Medical cannabis related adverse events after six months

**Adverse event**	**Number of patients (N=239)***
Dizziness	19 (7.9)
Dry mouth	16 (6.7)
Nausea and vomiting	13 (5.4)
Hyperactivity	12 (5.0)
Psychoactive	11 (5.2)
Drowsiness	10 (4.2)
Increased appetite	9 (3.8)
Weakness	8 (3.3)
Poor concentration and memory	6 (2.5)
Cough	5 (2.1)
Headache	5 (2.1)
Drop of sugar level	4 (1.7)
Hallucinations	3 (1.3)
confusion and disorientation	1 (0.4)
Tremor	1 (0.4)

*We included 211 who used cannabis at six months follow-up in addition to 28 patients who ceased cannabis before six months

Supplementary table 4. Symptom prevalence at intake and after six months

Symptoms	Change at 6 months			p value**
	**Symptom* Disappeared**	**Improvement***	**No change or Deterioration***	
Sleep problems, No. (%)	26 (13.5)	144 (75.0)	22 (11.4)	<0.001
Weakness, No. (%)	5 (4.9)	57 (56.4)	39 (38.6)	<0.001
Cognitive impairment, No. (%)	5 (21.7)	4 (17.4)	14 (60.9)	0.004
Spasticity, No. (%)	3 (2.4)	84 (69.4)	34 (28.0)	<0.001
Depression, No. (%)	2 (1.6)	101 (83.4)	18 (14.8)	<0.001
Anxiety, No. (%)	4 (3.5)	87 (77.0)	22 (19.4)	<0.001
Restlessness, No. (%)	6 (8.9)	58 (86.5)	3 (4.4)	<0.001
Dizziness, No. (%)	9 (12.7)	44 (62.0)	18 (25.3)	<0.001
Constipation, No. (%)	5 (8.3)	30 (50.0)	25 (41.6)	<0.001
Nausea, No. (%)	9 (17.0)	32 (60.4)	12 (22.6)	<0.001
Lack of appetite, No. (%)	12 (22.6)	34 (64.1)	7 (13.2)	<0.001
Drowsiness, No. (%)	1 (2.4)	26 (63.4)	14 (34.1)	<0.001

*Presented in percentage per symptom

**For symptom at intake vs. symptom improvement or disappearing at six months

Supplementary table 5. Changes in other drug regimens after six months of treatment with cannabis.

Drug class at baseline	Number of patients who stopped	Number of patients who reduced the dose	Number of patients who did not change the dose	Number of patients who initiate or increased the dose	Unknown	p value®
Benzodiazepines (n=118)	14 (11.8)	10 (8.4)	42 (35.6)	1 (0.8)	51 (43.2)	<0.001
Opioid analgesics* (n=126)	18 (14.3)	10 (7.9)	39 (30.9)	3 (2.3)	56 (44.4)	<0.001
SSRI or SNRI (n=77)	6 (7.7)	4 (5.1)	31 (40.2)	1 (1.2)	35 (45.4)	1.000
TCA (n=40)	12 (30.0)	0 (0.0)	12 (30.0)	1 (2.5)	15 (37.5)	0.021
Neuropathic pain drugs** (n=114)	17 (14.9)	10 (8.7)	40 (35.0)	3 (2.6)	44 (38.6)	<0.001
NSAID's (n=67)	7 (10.4)	3 (4.4)	16 (23.9)	2 (2.9)	39 (58.2)	0.001
OTC† (n=85)	10 (11.7)	3 (3.5)	20 (23.5)	1 (1.1)	51 (60.0)	0.092

®For using drug at baseline vs. those who stopped or reduced drug dosage at six months

* Includes: Morphine, Tramadol, Fentanyl, Oxycodone, Buprenorphine, Oxycodone-naloxone (Targin), Acetaminophen-Oxycodone (Percocet), Codeine-Caffeine-Paracetamol (Rokacet).

** Includes: Pregabalin, Gabapentin, Amitriptyline.

† Includes: Paracetamol andDipyrone

SSRI – Selective Serotonin Reuptake Inhibitor; SNRI – Serotonin–Norepinephrine Reuptake Inhibitor. NSAIDS's - Non-Steroidal Anti-Inflammatory Drugs, TCA - Tricyclic antidepressant

Supplementary table 6. Study outcomes stratified by primary vs. secondary fibromyalgia

	Primary Fibromyalgia			*Secondary Fibromyalgia*		
Variable	*Baseline*	*Six months*	*P value*	*Baseline*	*Six months*	*P value*
Pain intensity (0-10 VAS scale)	*9.0 (8.0-10.0)*	*5.0 (4.0-6.0)*	*<0.001*	*9.0 (8.0-10.0)*	*4.0 (3.0-6.0)*	*<0.001*
Quality of life (5 points Likert scale)	*2.0 (1.0-2.0)*	*4.0 (3.0-4.0)*	*<0.001*	*2.0 (1.0-2.25)*	*4.0 (4.0-4.0)*	*<0.001*
Multivariable Analysis	*Odds Ratio*	*95% C.I*	*P value*	*Odds Ratio*	*95% C.I*	*P value*
Age > 60 years	*0.42*	*0.18-1.02*	*0.06*	*0.10*	*0.01-1.00*	*0.05*
Concerns about cannabis - prior to treatment initiation	*0.41*	*0.16-1.04*	*0.06*	*0.22*	*0.04-1.36*	*0.10*
Spasticity	*2.65*	*1.12-6.25*	*0.03*	*1.59*	*0.31-8.13*	*0.57*
Previous experience with cannabis	*3.25*	*1.20-8.80*	*0.02*	*0.41*	*0.04-4.69*	*0.47*

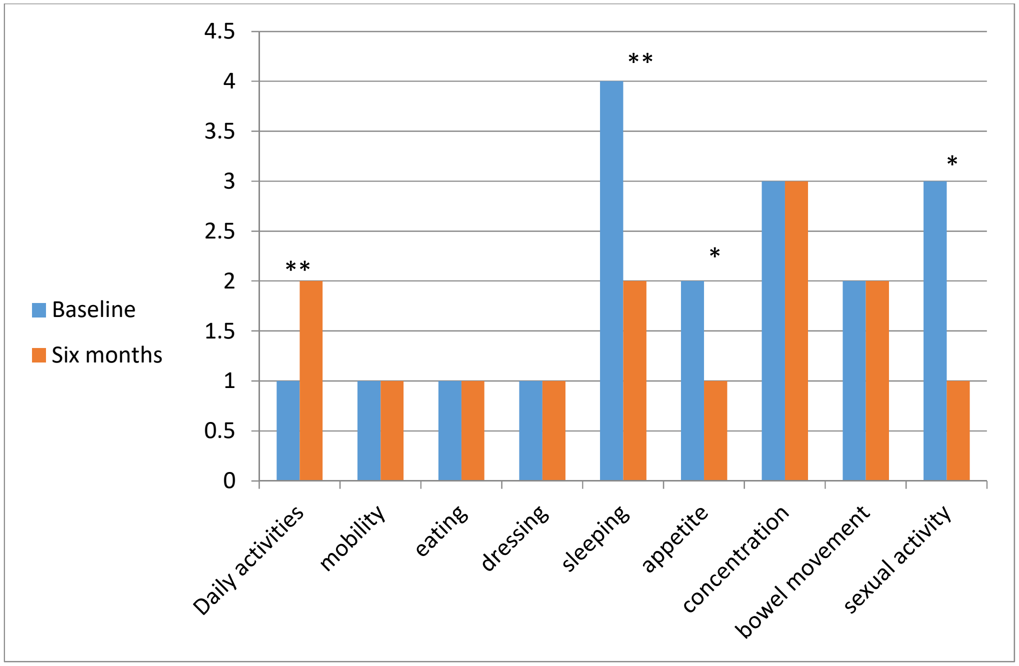

Supplementary Figure 1. Quality of life components on a 5-points Likert scale at baseline and at six months of follow-up. *$p<0.05$ **$p<0.001$.
